# Depsides: Lichen Metabolites Active against Hepatitis C Virus

**DOI:** 10.1371/journal.pone.0120405

**Published:** 2015-03-20

**Authors:** Thi Huyen Vu, Anne-Cécile Le Lamer, Claudia Lalli, Joël Boustie, Michel Samson, Françoise Lohézic-Le Dévéhat, Jacques Le Seyec

**Affiliations:** 1 CNRS, UMR-6226, Institut des Sciences Chimiques de Rennes (ISCR), Rennes, France; 2 Université de Rennes 1, Rennes, France; 3 Université de Toulouse III, Toulouse, France; 4 INSERM, UMR-1085, Institut de Recherche Santé Environnement & Travail (IRSET), Rennes, France; 5 Fédération de Recherche BioSit de Rennes, Rennes, France; Graz University of Technology (TU Graz), AUSTRIA

## Abstract

A thorough phytochemical study of *Stereocaulon evolutum* was conducted, for the isolation of structurally related atranorin derivatives. Indeed, pilot experiments suggested that atranorin (1), the main metabolite of this lichen, would interfere with the lifecycle of hepatitis C virus (HCV). Eight compounds, including one reported for the first time (2), were isolated and characterized. Two analogs (5, 6) were also synthesized, to enlarge the panel of atranorin-related structures. Most of these compounds were active against HCV, with a half-maximal inhibitory concentration of about 10 to 70 µM, with depsides more potent than monoaromatic phenols. The most effective inhibitors (1, 5 and 6) were then added at different steps of the HCV lifecycle. Interestingly, atranorin (1), bearing an aldehyde function at C-3, inhibited only viral entry, whereas the synthetic compounds 5 and 6, bearing a hydroxymethyl and a methyl function, respectively, at C-3 interfered with viral replication.

## Introduction

Hepatitis C virus (HCV) is a small, enveloped virus of genus *Hepacivirus*, from the Flaviviridae family. About 170 million people worldwide are chronically infected with HCV, and there are three to four million new infections each year [1], principally through blood transmission. The high genetic variability of the virus has hindered vaccine development. However, new treatments have emerged in the last few years, with the discovery of direct-acting antivirals (DAAs), which were added to the previous standard care procedure based on the use of pegylated interferon (IFN) and ribavirin (RVB). Clinical trials currently underway suggest that IFN-free regimens combining several DAAs will cure almost all cases of hepatitis C, making it possible to avoid the severe adverse effects of IFN treatment [2]. Nevertheless, the current high cost of such treatments may restrict them to only the wealthiest patients and nations [3]. There is, therefore, still a need to develop alternative and complementary approaches to treatment for a large proportion of patients.

It has recently been shown that natural compounds may be a source of anti-HCV drugs [4,5], but the presence of such active molecules has never been investigated in lichens. Lichens are symbiotic associations between at least one fungal and an algal and/or cyanobacterial partner. Some 1,035 secondary metabolites have been isolated from the 18,500 lichen species from the Arctic to tropical habitats described to date, and many more compounds remain to be characterized [6]. Most of these compounds are polyketides, polyphenols, quinones or terpenoids, presumably of fungal origin [6,7], and their biological activities remain largely underexplored. However, a few have been shown to have antibiotic, antimycobacterial, anti-inflammatory, analgesic, antipyretic, or antiproliferative effects [7]. Antiviral activities have also been reported for some secondary lichen metabolites, such as (+)-usnic acid, sekikaic acid and anthraquinones, targeting Junin and Tacaribe arenaviruses [8], respiratory syncytial virus [9] and herpes simplex virus type 1 [8], respectively. In a pilot study, we discovered that atranorin seemed to inhibit HCV. As this depside is a major compound of the genus *Stereocaulon*, we conducted a phytochemical investigation on a crude extract of *S*. *evolutum*. Seven structurally related atranorin derivatives were isolated, together with seven other secondary metabolites. Two atranorin analogs were also synthesized. The relationship between structure and antiviral activity against HCV was discussed with these molecules.

## Materials and Methods

### General procedures

All solvents for chromatography were purchased from Sigma-Aldrich (France). Thin-layer chromatography (TLC) was carried out on silica gel plates (Merck silica gel 60F_254_) with the B, C, and G standard solvent systems for the identification of substances from lichens [10]. The spots were first visualized under UV light and then after spraying with anisaldehyde–H_2_SO_4_ reagent. Circular chromatography and column chromatography (CC) were carried out on silica gel (40–63 μm, Kieselgel 60, Merck, 7667). Medium-pressure liquid chromatography (MPLC) was conducted on a SPOT Liquid Chromatography Flash apparatus (Armen Instrument), using prepacked silica gel or RP-18 columns (Chromabond, Merck). ^1^H, ^13^C and 2D NMR spectra were recorded on a BrukerAvance III400 or a Bruker 300 spectrometer with deuterated solvents. High-resolution mass spectrometry (HR-MS) measurements were carried out to determine exact mass, on a Bruker Maxis 4G, MicrO-Tof Q 2, a Thermo-fisher Q-Exactive or a Waters Q-Tof 2 mass spectrometer for chemical ionization. HPLC-ESI and ESI-MS_n_ mass spectra were obtained with a LCQ Deca ion trap mass spectrometer (Thermo Finnigan) equipped with an ESI source, in negative ionization mode. The Ion Trap MS system was coupled to an HPLC system, including a Surveyor autosampler (Thermo Finnigan), a 1100 Series binary pump (Agilent Technologies), a UV-6000 DAD UV–visible detector (Thermo Finnigan) and a Zorbax Eclipse XDB-C18 column (150 mm x 2.1 mm, Agilent Technologies) maintained at 30°C by a thermostat.

### Ethics statement

Specimens of *S*. *evolutum* Graewe were collected from siliceous rocks in Saint Just (Ille et Vilaine, France), by F. Le Dévéhat, in November 2011. No specific permits were required for the described field studies in Saint Just (Ille et Vilaine). The research sites are not privately owned or protected in any way and field studies did not involve endangered or protected species. A voucher specimen (JB/10/121) has been deposited in the Herbarium of the Department of Pharmacognosy and Mycology of the University of Rennes 1 (France).

### Extraction and isolation

Air-dried thalli of the lichen *S*. *evolutum* Graewe (300 g) were successively extracted with *n*-hexane, acetone and tetrahydrofuran, by maceration for one day at room temperature (3 times x 2 L). A pure white compound **1** (6.2 g) was precipitated from the *n*-hexane and acetone extracts by evaporating off the solvents at room temperature. The *n*-hexane filtrate (1.55 g) was fractionated on successive silica gel columns and circular chromatography, to obtain compounds **15** (7.0 mg), **16** (8.5 mg) and **17** (23.3 mg). The acetone filtrate was concentrated down to a volume of 50 mL under vacuum and filtered, to yield a white precipitate P1 (1.5 g) and a soluble portion S1 (4.8 g). The P1 fraction was purified on an MPLCRP-18 column and subjected to precipitation steps, to yield compounds **11** (25.5 mg) and **13** (1.2 g). The acetone filtrate S1 (4.8 g) was subjected to repeated MPLC C18 and silica gel MPLC or column chromatography to isolate compounds **4** (5.2 mg), **7** (159 mg), **8** (6.5 mg), **9** (9.9 mg), **14** (5 mg), **10** (14.5 mg), **12** (7.2 mg), **2** (8.4 mg), and **3** (20,9 mg). Detailed protocols are available in Supporting Information (S1 Protocol).

### Detection of compound 2 by LC-ESI-MS/MS in acetone and ethyl acetate extracts

Dried ground *S*. *evolutum* powder (1 g) was macerated at room temperature in acetone or ethyl acetate (15 mL) for 24 h. This extraction process was repeated three times. Simultaneously, pure atranorin (1 g) or a dried ethyl acetate extract (100 mg) was macerated in acetone (15 mL or 1.5 mL) for one week. Compound **2** was detected in extracts by HPLC-ESI, as previously described [11].

### Hemisynthesis of atranorin derivatives

Methyl-8-hydroxy-4-*O*-demethylbarbatate (**5**): a solution of atranorin (93.5 mg, 0.25 mmol) in methanol (10 mL, 0.025 M) was stirred at 0°C for a few minutes. Sodium borohydride (18.9 mg, 0.5 mmol) was then added and the mixture was stirred for a further 1 h at room temperature. The reaction was quenched by adding a few drops of saturated ammonium chloride. The solvent was evaporated off and the residue was purified on a small column of silica gel and eluted with a solvent system of petroleum ether-CH_2_Cl_2_ (1:9), followed by CH_2_Cl_2_ (100%). Compound **5** (79.3 mg, 84%) was obtained as a white crystalline solid [10,12].

Methyl-4-*O*-demethylbarbatate (**6**): a solution of methyl-2,4-dihydroxy-3,6-dimethylbenzoate (compound **7**) (147 mg, 0.75 mmol) in anhydrous CH_2_Cl_2_ (3.75 mL, 0.2 M) was added dropwise, at-70°C, to a 1.0 M solution of boron tribromide in anhydrous CH_2_Cl_2_ (3 mL, 3 mmol). The reaction mixture was stirred for 12 h at room temperature. It was then hydrolyzed with 10 mL of 2 N HCl solution and subjected to extraction with ethyl acetate. The organic phase was washed with 2 N HCl and then with a saturated NaCl solution, dried over MgSO_4_, filtered and evaporated. The residue was passed through a silica gel column, with 20% ethyl acetate-petroleum ether as the eluent. β-orcinol carboxylic acid (103.1 mg, 74%) was obtained as a white crystalline solid [10]. Trifluoroacetic anhydride (0.5 mL, 8 mmol) was added to a solution of β-orcinol carboxylic acid (98.3 mg, 0.54 mmol) in anhydrous diethyl ether (5 mL, 0.1 M) under nitrogen, and the mixture was stirred for ten minutes at room temperature. We then added compound **7** (88.2 mg, 0.45 mmol) and the mixture was stirred for 24 h at room temperature. The solvent was removed under low pressure and the residue was purified by chromatography on deactivated silica gel with 30% ethyl acetate/*n*-hexane, with a gradual increase in polarity to 50% ethyl acetate/*n*-hexane. Methyl-4-*O*-demethylbarbatate (**6**) was obtained as a white solid (24 mg, 15%) [13].

### Cell culture

Huh-7.5.1 cells [14] were maintained in Dulbecco's modified Eagle's medium (DMEM) containing 4.5 g/l D-glucose, 4 mM L-glutamine (Life Technologies), supplemented with 100 units/mL penicillin (Life Technologies), 100 μg/mL streptomycin (Life Technologies), non-essential amino acids (Sigma-Aldrich), 1 mM Hepes (Life Technologies) and 10% heat-inactivated fetal calf serum (FCS, Hyclone). Lichen compounds and control HCV inhibitors (erlotinib from Abcam and telaprevir from Euromedex) were dissolved in dimethyl sulfoxide (DMSO). In cell viability and HCV *in vitro* propagation experiments, the final concentration of DMSO was systematically adjusted to 0.1%. We checked that this amount of DMSO had no effect on the biological cycle of HCVcc *in vitro* (data not shown).

### Virus production and titration

HCVcc was generated from the FL-J6/JFH-5’C19Rluc2AUbi construct, a monocistronic, full-length HCV genome that expresses *Renilla* luciferase [15]. It was produced and titrated as described elsewhere [16,17], except for the readout for luciferase activity measurement. Cell viability was evaluated with the Cell Proliferation Reagent WST-1 (Roche), according to the manufacturer’s instructions. Luciferase assays were performed according to the manufacturer’s instructions (Promega) and measurements were performed on a Centro XS3 LB960 luminometer (Berthold Technologies).

### Data analysis

Cell viability and viral replication data from at least three independent experiments were analyzed. The IC_50_ values of the compounds were determined from dose-response curves generated with seven to 12 concentrations of each compound. Curves were fitted to the data points by nonlinear regression analysis and IC_50_ values were interpolated from the resulting curves with GraphPad Prism 5.00 software.

## Results and Discussion

### Phytochemical composition

**Fig 1:**
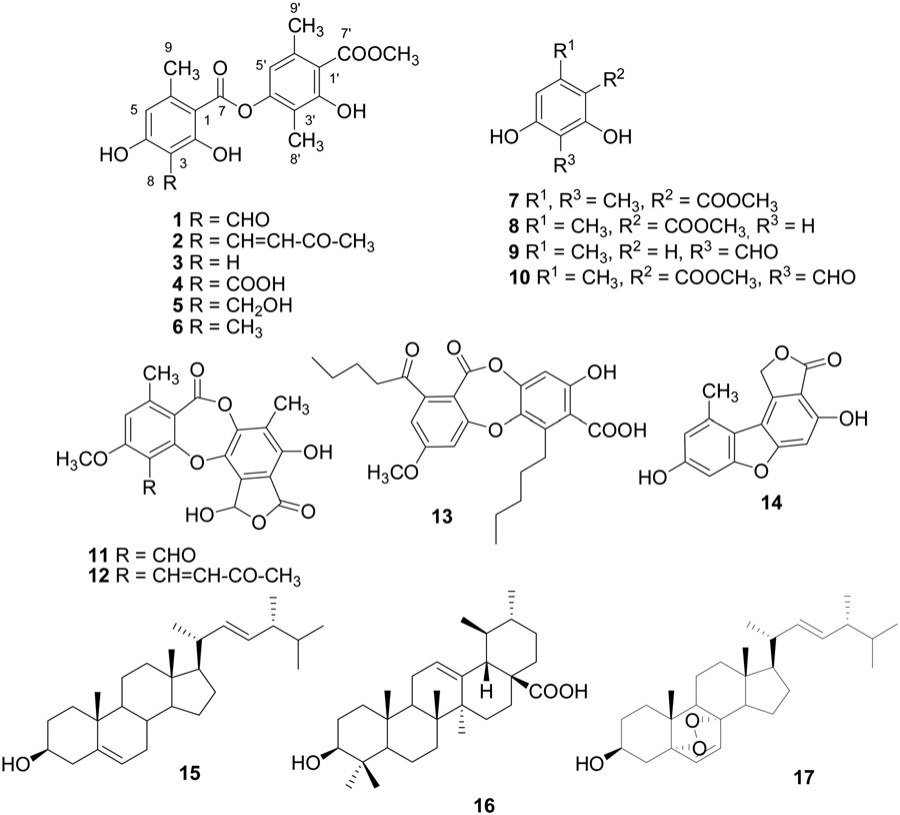
Structures of compounds isolated from the lichen *S. evolutum* and of the semi-synthetic compounds 5–6.

Phytochemical investigations were carried out on whole thalli of *S*. *evolutum*, with the aim of isolating metabolites structurally similar to atranorin. Briefly, extractions with solvents of increasing polarity (*n*-hexane, acetone, tetrahydrofuran) yielded three fractions, which were purified by extensive and repeated column chromatography on silica gel and RP-18, to give 15 compounds (Fig. 1). The structure of the new compound **2** was assigned by detailed spectroscopy and the structures of the known compounds were determined by comparisons with published data.

Eight atranorin derivatives (**1**–**4** and **7**–**10**) were isolated in addition to three depsidones (stictic acid **11**, isidiophorin **12**, lobaric acid **13** [18]), one dibenzofurane (strepsilin **14** [19]) and three terpenoids (brassicasterol **15** [20], ursolic acid **16** [10], ergosterol peroxide **17** [21]).

**Fig 2:**
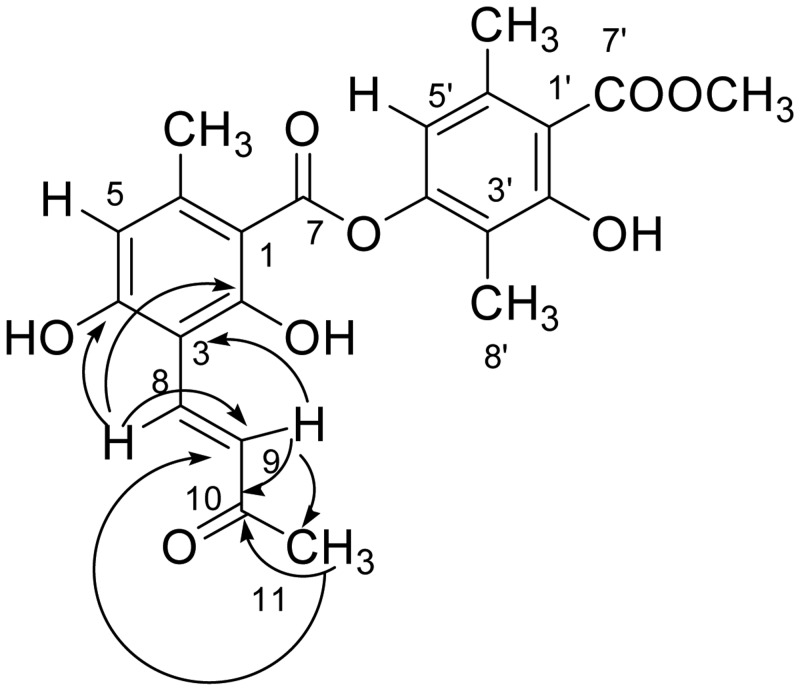
HMBC key correlations for compound 2.

Compound **2** (a white amorphous powder) was found to have a molecular formula of C_22_H_22_O_8_ on negative HRESIMS (m/z = 413.1237 [M-H]^-^), with 11 degrees of unsaturation. Analysis of the ^1^H NMR, ^13^C NMR (Table 1) and HMQC spectra (S1 Fig.) revealed that compound **2** was very similar to atranorin (compound **1**), except that it lacked the aldehyde signal and had additional signals for a ketone group at *δ*_C_ 198.6 (C-10), a methyl group at *δ*_H_ 2.28 (3H, s, H-11) and *δ*_C_ 27.5, and two conjugated aromatic protons at 7.18 (1H, d, *J* = 16.5 Hz, H-9) and 7.85 (1H, d, *J* = 16.5 Hz, H-8), indicating the presence of a *trans* double bond (*δ*_C_ 128.7 and 133.1). Extensive analysis of the HMBC spectrum (Fig. 2) indicated that the protons of the methyl group at *δ*_H_ 2.28 were correlated with C-10 (*δ*_C_ 198.6) and C-9 (*δ*_C_ 128.7). Moreover, H-9 (*δ*_H_ 7.18) and H-8 (*δ*_H_ 7.85) were correlated with the C-10 carbonyl group. These data suggest the presence of a methylvinyl ketone chain. Finally, HMBC correlations from proton H-9 (*δ*_H_ 7.18) to C-3 (*δ*_C_ 107.6), together with correlations from H-8 (*δ*_H_ 7.85) to C-2 (*δ*_C_ 163.7) and C-4 (*δ*_C_ 162.6) (Table 1) indicated that compound **2** was an atranorin derivative with a methylvinyl ketone chain substitution at position C-3. Accordingly, compound **2** was formulated as shown (Fig. 2) and was designated (*E*)-[3-hydroxy-4-(methoxycarbonyl)-2,5-dimethylphenyl]-2,4-dihydroxy-6-methyl-3-(3-oxobut-1-en-yl) benzoate.

**Table 1:** NMR data for compound 2 in DMSO-*d*_6_ (400 MHz for ^1^H and 100 MHz for ^13^C).

Position	δ_H_	δ_C_
1		104.2
2		163.7
3		107.6
4		162.6
5	6.50(s)	111.7
6		144.3
7		169.1
8	7.85 (d, 16.5)	133.1
9	7.18 (d, 16.5)	128.7
10		198.6
11	2.28(s)	27.5
12	2.56(s)	23.9
1’		115.5
2’		157.2
3’		116.0
4’		150.7
5’	6.76 (s)	115.7
6’		136.4
7’		169.5
8’	1.99 (s)	9.2
9’	2.34 (s)	20.9
OCH_3_	3.89 (s)	52.2
2’-OH	10.52 (s)	
4-OH	11.50 (brs)	
2-OH	12.04 (s)	

**Fig 3:**
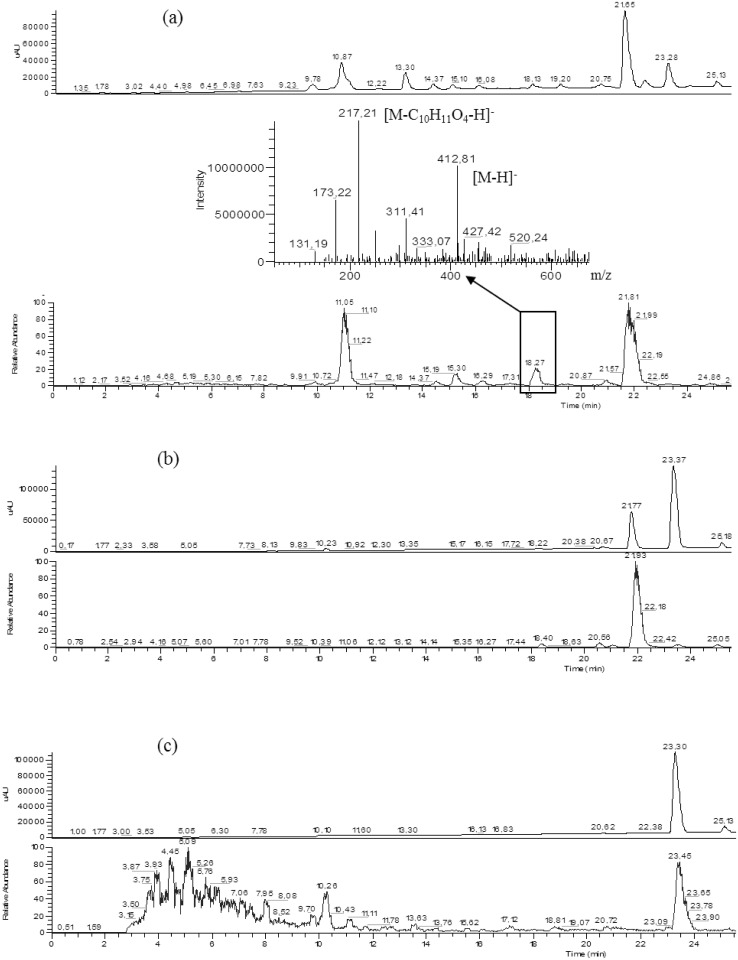
LC-MS analysis. Compound **2** (extracted at Rt = 19.48 min) was detected through PDA chromatograms (total scan at λ = 220–600 nm), base-peak mass chromatograms and MS spectra of extemporaneously prepared acetone extract of *S*. *evolutum* (**a**), an ethyl acetate extract of *S*. *evolutum* (**b**) and pure atranorin macerated in acetone for one week (**c**).

The structural similarity between compound **2** and atranorin suggested that this compound might be produced during the maceration of *S*. *evolutum* in acetone. We tested this hypothesis, by performing LC-MS analysis on freshly prepared crude acetone and ethyl acetate extracts of *S*. *evolutum*. Compound **2** was found in the crude acetone extract, but was not detected in the ethyl acetate extract (Fig. 3). Moreover, atranorin and the crude ethyl acetate extract were macerated for one week in acetone without the detection of even trace amounts of **2**, indicating that the formation of compound **2** in acetone required the lichen to be present. Stictic acid and its methylvinyl ketone derivative, isidiophorin, were isolated from the acetone extract. These two compounds have been consistently reported to be isolated simultaneously in the acetone fraction [22–24]. This suggests that, like compound **2**, isidiophorin was probably derived from stictic acid.

Two newly reported depsides from the genus *Stereocaulon*, methyl-3’-methyllecanorate (**3**) and cladonioidesin (**4**) [25], were also identified. Interestingly, these depsides and compound **2** differed from atranorin (**1**) in terms of their C-3 substitutions. Indeed, the substituent on C-3 consisted of an aldehyde group in **1**, a methylvinyl ketone moiety in **2**, a proton in **3** and a carboxylic group in **4**. We therefore synthesized two analogs, one bearing a methyl alcohol group and the other a methyl group (**5** and **6** respectively) on C-3, to evaluate the influence of the degree of oxidation of the C-3 substituent on HCV inhibition. The synthesis of these molecules was based on the protocol of Pulgarin [26]. Reduction of the CHO group of atranorin generated compound **5,** and esterification of β-orcinol carboxylic acid with compound **7** resulted in compound **6**. Finally, four monoaromatic phenols (methyl- β-orcinol carboxylate (**7**), methyl orsellinate (**8**) [10], atranol (**9**) and methyl haematommate (**10)** [10]), were also isolated.

Thus, the phytochemical investigation of *Stereocaulon evolutum* extracts and the synthesis of two derivatives yielded a panel of 10 atranorin derivatives for evaluation on hepatitis C virus.

### Antiviral activity of atranorin derivatives

We evaluated the anti-HCV activity of the 10 atranorin-related compounds, using a virus grown in cell culture (HCVcc) with a modification of its genome resulting in expression of the *Renilla* luciferase reporter gene [15]. Two control inhibitors, erlotinib and telaprevir, were systematically included in each experiment. These two compounds are known to interfere with the entry and replication steps of viral cycle, respectively. The maximum dose of each tested compound was 100 μM, because most of these molecules became insoluble in aqueous solution at higher concentrations. The first experimental procedure used involved the preincubation of human hepatic Huh-7.5.1 cells for 1 h with various doses of the molecules. The cells were then cultured in the presence of both the molecules tested and the virus, for an additional 48 h. At the end of the incubation period, we assessed cell viability and viral replication. This protocol made it possible to test the effects of molecules on both virus entry and replication. Initial observations indicated that most compounds were not cytotoxic (Table 2 and S2 Fig.). For compounds **1**, **4**, **8**, **9** and **10**, high doses led to a minimal loss of signal, due to either weak toxicity or an antiproliferative effect similar to that observed for erlotinib, an epidermal growth factor receptor inhibitor. Conversely, compounds **3** and **5** were more toxic, with estimated half-maximal cytotoxicity concentrations (CC_50_) of 82 μM and 106 μM, respectively (Table 2). Remarkably, compound **6** tended to give a slightly, but reproducibly higher viability signal when used at high doses, with a peak at about 50 μM. This probably reflects a modest stimulation of cell proliferation, an effect due solely to the lichen metabolite, because a similar profile was observed in the absence of viral infection (data not shown). At concentrations greater than 50 μM, cytotoxic effects emerged, as shown by the decrease of the viability signal.

**Table 2:** Anti-HCV activity and toxicity of atranorin derivatives^a^.

			Viability at			
	Compounds	IC_50_ (μM)	50 μM	100 μM	CC_50_ (μM)	SI
**Depsides**	**1**	22.3 ±8.0	95.9 ±4.5	91.8 ±5.9	>100	-
	**2**	64.5 ±26.5	110.2 ±5.9	107.4 ±5.7	>100	-
	**3**	25.4 ±1.7	52.7 ±0.6	63.1 ±2.9	81.9 ±13.6	3.2
	**4**	NI	104.0 ±2.5	90.4 ±3.4	>100	-
	**5**	11.8 ±1.1	88.4 ±7.3	55.8 ±5.9	105.8 ±7.3	8.9
	**6**	13.3 ±1.6	144.7 ±3.2	81.6 ±20.6	>100	-
**Monoaromatic**	**7**	50.6 ±5.0	101.2 ±5.6	97.3 ±6.2	>100	-
**phenols**	**8**	≥ 100	105.5 ±2.9	93.4 ±4.2	>100	-
	**9**	40.3 ±0.6	91.1 ±3.5	78.7 ±2.3	>100	-
	**10**	55.5 ±7.9	88.1 ±5.5	72.2 ±2.0	>100	-
			**5 μM**	**10 μM**		
**Controls**	Telaprevir	0.18 ±0.02	101.9 ±0.9	94.1 ±4.0	-	-
	Erlotinib	0.64 ±0.18	96.5 ±4.7	86.5 ±5.1	-	-

^a^Compounds were included in antiviral activity and viability assays based on the infection of Huh-7.5.1 cells with HCVcc. The results presented are the means ± SEM from three sets of experiments. NI, no inhibition. For compounds found to be cytotoxic at the doses tested, we determined the CC_50_ and SI (selectivity index).

With the exception of cladonioidesin (**4**), all the atranorin derivatives inhibited HCV in a dose-dependent manner. Thus, the half-maximal inhibitory concentrations (IC_50_) of lichen derivatives ranged between about 10 and 70 μM, whereas those of the controls were consistent with published values [27–29] (Table 2). The IC_50_ values of these lichen metabolites were similar to those published for other natural molecules [4]. The high standard error of the mean (SEM) observed for the IC_50_ of compound **2** probably resulted from the observed instability of the molecule in solution, even if dissolved immediately before use. Monoaromatic phenols and depsides had different IC_50_ values (Table 2). With the exception of compound **2,** which was instable, depsides appeared to be more active than monoaromatic phenols, and this finding was confirmed statistically by an analysis of variance (Table 2, S1 Table). The lipophilicity of these lichen metabolites may govern their efficacy in the targeted cells, by influencing their passive diffusion across the cell membrane, and thus their intracellular bioavailability. We used ALOGPS 2.1 freeware to predict the theoretical partition coefficient (logP) of the studied atranorin derivatives, to obtain an index of lipophilicity. Depsides, which had higher levels of antiviral activity, were also more lipophilic than monoaromatic phenols (S3 Fig.). By contrast, lipophilicity was not a discriminant parameter within the depside family tested. However, our results highlight the importance of the nature of the chemical group at position C-3 in depsides for their anti-HCV activity, as these molecules have identical chemical structures except for this position. The most active depsides (**1**, R = CHO, IC_50_ = 22.3 μM; **5**, R = CH_2_OH, IC_50_ = 11.8 μM and **6,** R = CH_3_, IC_50_ = 13.3 μM) were selected for additional investigations. We excluded molecule **3** from further analysis because its selectivity index (SI, ratio of CC_50_ to IC_50_) was below that of compound **5** (Table 2).

### Targeted stages of the HCV life cycle

**Fig 4:**
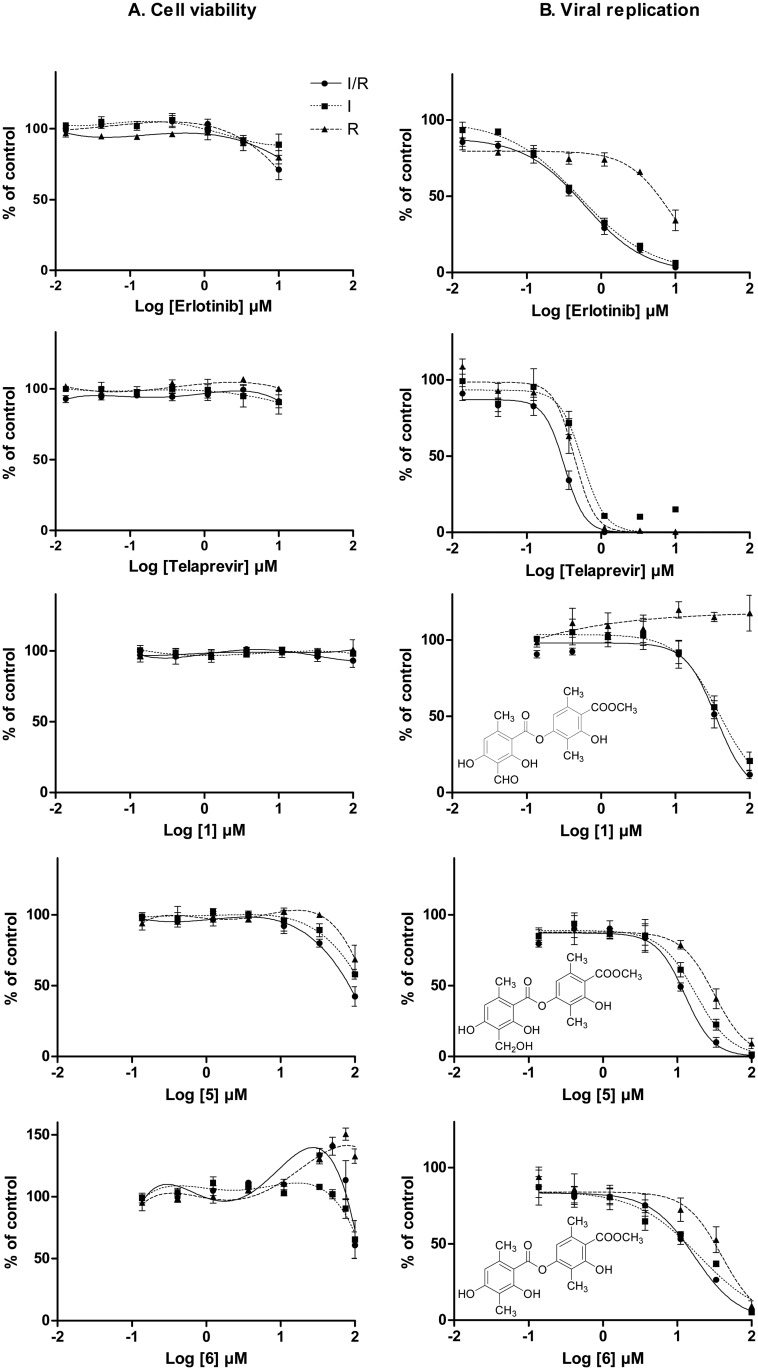
Effects of compounds 1, 5 and 6 on cell viability and HCV propagation *in vitro*. One day after seeding at 50,000 cells/cm^2^, Huh-7.5.1 cells were infected with HCVcc at a MOI ~ 0.03 for 17 h (inoculation phase: I). The inoculum was removed and cell cultures were washed before the addition of new medium and incubation for an additional 30 h (replication phase: R). Compounds were present at various concentrations either during both phases (I/R: black circles and solid lines) or during only one phase (I: black squares and dotted lines; R: black triangles and dashed lines). (**A**) Cell viability and (**B**) viral replication were assessed at the end of the incubation period. Results are expressed as percentages (mean ± SEM; *n* = 3) of the mean values obtained for the respective control cultures, in which cells were incubated with vehicle (0.1% DMSO) alone. Erlotinib and telaprevir were used as positive antiviral drug controls inhibiting the entry and replication steps, respectively.

An experimental procedure was designed to determine the inhibitor class (entry or replication) to which compounds **1**, **5** and **6** belonged. HCVcc were added to the cell culture during a limited time window (around 17 h). The cells were then washed to eliminate viruses from the inoculum and viral replication was allowed to take place for an additional 30 h. The selected compounds were present in the culture medium during inoculation (I), replication (R), or both these phases (inoculation and replication, I/R). The reference drugs and compound **1** had no effect on cell viability, regardless of the incubation conditions (Fig. 4A). A small anti-proliferative effect was detectable only with high doses of erlotinib. The cytotoxic effect of compound **5** was also reproduced in these new conditions, with a similar CC_50_ (~ 100 μM). Periods of more than 17 h (R and I/R) of incubation with compound **6** resulted in an enhancement of the cell viability signal over a narrow range of high concentrations, probably reflecting the capacity of this molecule to induce cell proliferation, as reported above. At a concentration of 100 μM, this compound induced some cytotoxicity, as demonstrated by a decrease in signal intensity, except in R conditions, in which the toxic effect was shifted to higher concentrations. In terms of antiviral activity, we expected to obtain two inhibition profiles for the luminescence signal, depending on the step of the viral lifecycle targeted by the drug (Fig. 4B). As shown with erlotinib, inhibitors of viral entry are most efficient if present at least during the initial interaction between cells and viruses (conditions I/R and I). The inhibitory effect on the establishment of replication (R conditions) observed at high doses was probably due principally to the inhibition of new infection cycles initiated by virions produced by cells infected with the inoculum. A different profile was obtained for telaprevir. This antiviral drug specifically targets the HCV protease, accounting for its inhibitory action regardless of the experimental conditions (I or R). Its presence in the medium from inoculation onwards prevented the priming of the infection process at the viral polyprotein maturation step, which normally takes place after the translation of the HCV genome issued from the infecting virus. This maturation step is also repeated during the replication phase, for amplification of the viral genome and virion assembly. It is therefore not surprising that inhibition was strongest when telaprevir was present throughout the viral lifecycle (I/R), as there was probably an additive effect. Surprisingly, differences were observed between the lichen metabolites tested. Compounds **5** and **6** had profiles typical of replication inhibitors, whereas compound **1** had effects similar to those of erlotinib (Fig. 4B). However, no inhibitory effect was observed when compound **1** was present in the culture medium only during inoculation. Had compound **1** been soluble enough to allow the evaluation of higher concentrations, its presence during the inoculation stage would probably have led to inhibition, as for erlotinib. These data again highlight the importance of the nature of the chemical moiety at C-3 in depsides for their anti-HCV activity. The chemical group in this position seems to influence not only the potency of the molecule, but also its mechanism of action. Further studies are required to identify the processes involved because, for example, we cannot rule out a role in the inhibition of viral entry for compounds **5** and **6,** even though they appeared to act as replication inhibitors in this experiment. It could be hypothesized that the replacement of a methyl or hydroxymethyl by an aldehyde group in position C-3 would lead to a loss of replication inhibition, but maintenance of the ability to inhibit viral entry.

We show here, for the first time that compounds from lichens, such as atranorin, may display antiviral activity against HCV. Atranorin has already been described as an antioxidant [30], antinociceptive [31], antimalarial [32] and anti-inflammatory [33,34] compound. Our findings reveal that this molecule and some of its derivatives are potential new lead compounds for the development of anti-HCV drugs. Further studies of the antiviral properties of these molecules are required, but it is already known that atranorin is a common secondary metabolite of lichens, available in great amounts and easy to purify from lichens with a worldwide distribution. The next step will be determining the mechanisms of action of these molecules, which may act directly on viral particles (virucidal effect) or indirectly through effects on viral or cellular factors involved in HCV propagation. Evaluations of the spectrum of activity of these molecules against the various viral genotypes will also be required. It will also be important to determine the capacity of these molecules to limit the emergence of mutant resistant viruses to assess their therapeutic potential. Such studies may also provide information about both the mechanism of action of these molecules and the viral factor targeted. Multitherapy approaches are required to treat hepatitis C [2], so studies exploring the combination of these molecules with the various existing drugs will be required, to determine whether particular combinations have additive, synergistic or antagonistic effects. Despite the tremendous progress in treatment achieved in recent years with the advent of DAAs, studies are continuing and new antiviral drugs are still being discovered [35–39]. For instance, new candidate drugs with different mechanisms of action are currently being investigated [40]. These drugs will make it possible to diversify the treatments on offer, to cut costs, to improve resistance control and to resolve the remaining difficult-to-treat cases, particularly those in non-responders with advanced fibrosis and re-infected transplant patients. This research falls within the scope of efforts to find affordable natural anti-HCV compounds for the treatment of hepatitis C in developing countries, where this disease remains a major public health problem.

## Supporting Information

S1 Fig NMR spectra of compound 2. (DOCX)

S2 Fig Effects of ten atranorin-derivatives on cell viability. One day after seeding at 50,000 cells/cm^2^, Huh-7.5.1 cells were incubated 48 h with HCVcc at a MOI ~ 0.03 in the presence of various concentrations of the tested compounds which were already added to cell culture 1 h before. Cell viability and viral replication were assessed at the end of the incubation periods. Results for cell viability are expressed as percentages (mean ± SEM; *n* = 3) of the mean values obtained for the control culture, in which cells were incubated with vehicle (0.1% DMSO) alone. Erlotinib and telaprevir were used as positive antiviral drug controls inhibiting the entry and replication steps, respectively. Results for viral replication are presented in Table 2 in the main manuscript as IC_50_ mean values ± SEM.
(DOC)

S3 Fig Relationship between antiviral activity and theoretical partition-coefficients of lichen compounds. One day after seeding at 50,000 cells/cm^2^, Huh-7.5.1 cells were incubated 48 h with HCVcc at a MOI ~ 0.03 in the presence of various concentrations of the tested compounds which were already added to cell culture 1 h before. Viral replication was assessed at the end of the incubation periods to determine the respective IC_50_ (mean ± SEM, *n* = 3, see Table 2 in the main manuscript) of each compounds. The antiviral activities (IC_50_) of lichen metabolites (depsides in gray circles and monoaromatic phenols in open circles) were represented according to their theoretical partition-coefficients (logP) predicted with the free software ALOGPS 2.1. For clarity, three lichen metabolites were excluded from the analysis: compound **2** for its inaccurate IC_50_ value due to its instability, and the inactive compounds **4** and **8**.
(DOC)

S1 Protocol Detailed protocol for extraction and isolation of lichen metabolites. (DOC)

S1 Table Statistical comparison of anti-HCV activity of lichen metabolites (DOCX)
