# Supplementary material for: Depsides: Lichen Metabolites Active against Hepatitis C Virus
Source: PLoS One. 2015 Mar 20;10(3):e0120405. doi: 10.1371/journal.pone.0120405 (PMC4368788; doi:10.1371/journal.pone.0120405)
Supplement: S1 Fig — (DOCX) [file pone.0120405.s001.docx]

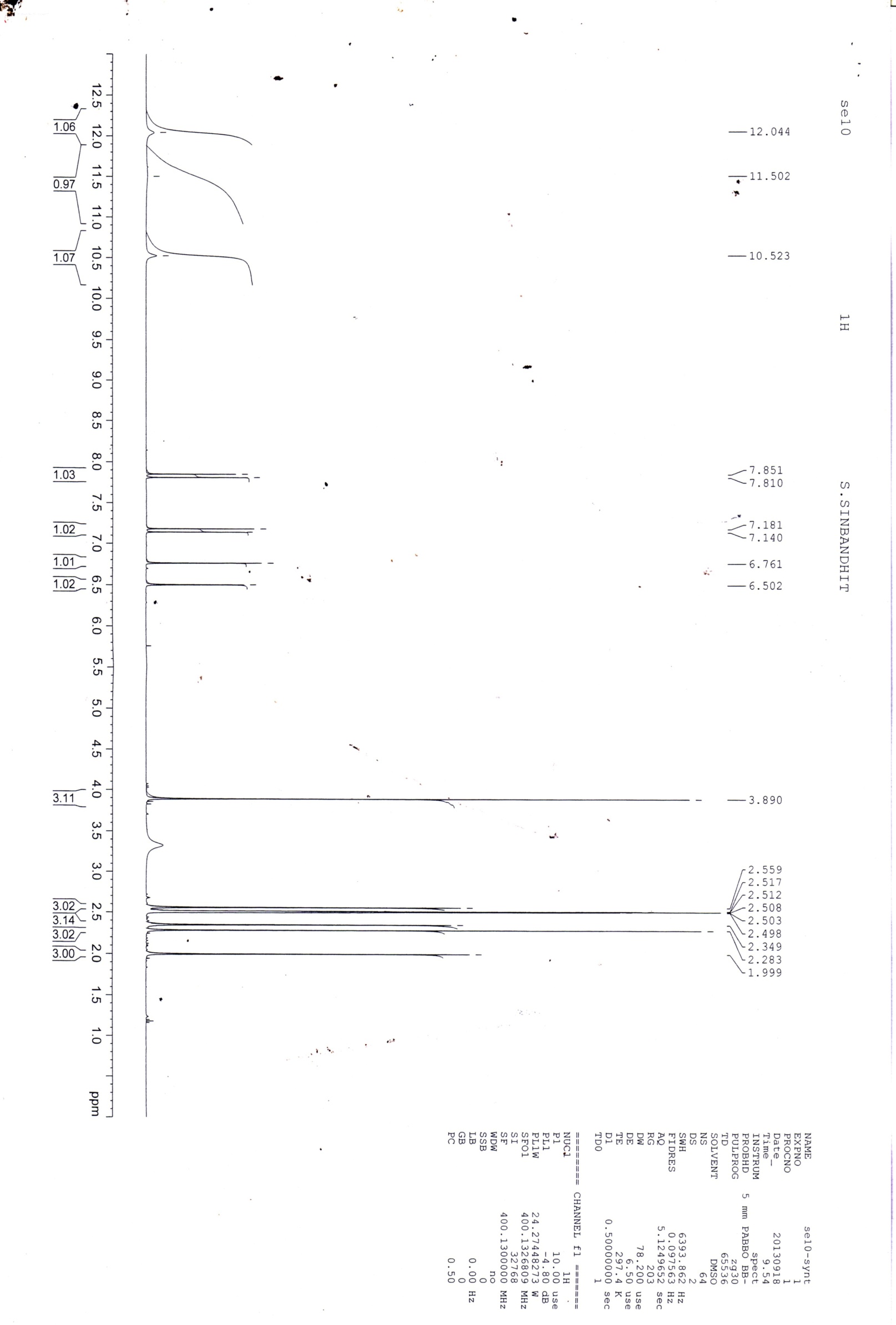

**Figure S1A** ^1^H NMR spectrum of compound **2** (400 MHz, DMSO-*d*_6_)


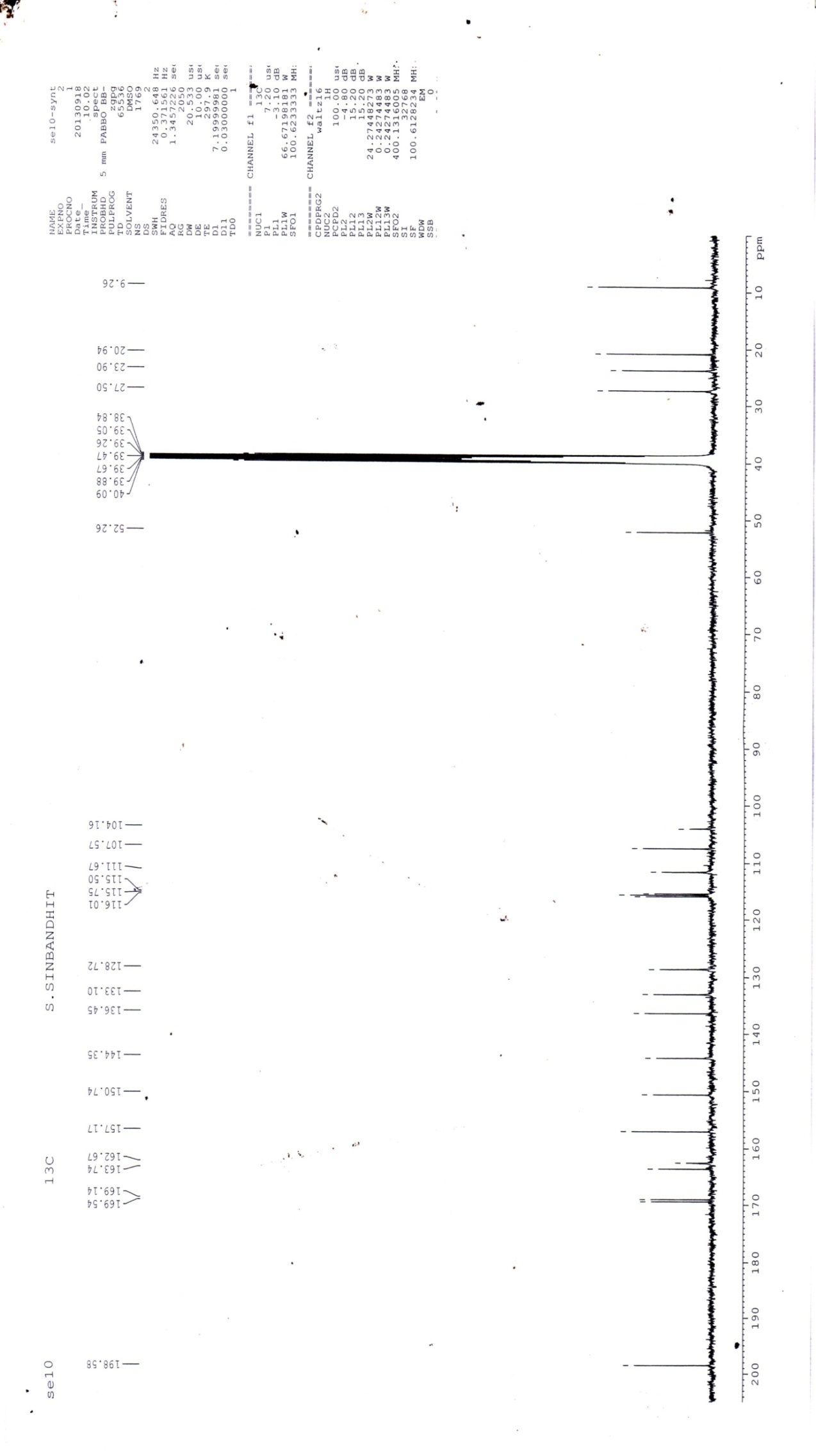

C-11

C-8’

C-9’

C-12

C-11

OCH_3_

**Figure S1B** ^13^C NMR spectrum of compound **2** (400 MHz, DMSO-*d*_6_)


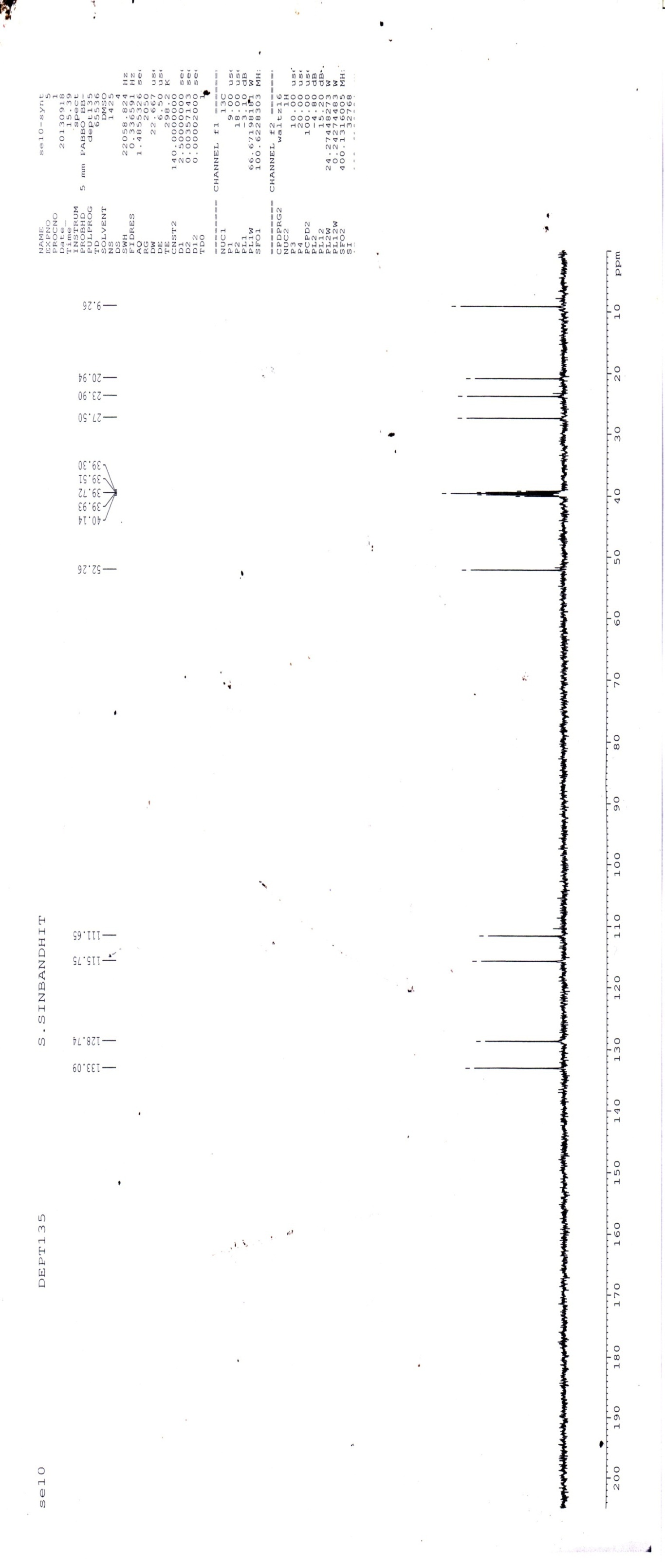


C-5

C-8’

C-9’

C-12

C-11

OCH_3_-7’

C-5’

C-9

C-8

**Figure S1C** ^13^C-DEPT135 spectrum of compound **2**


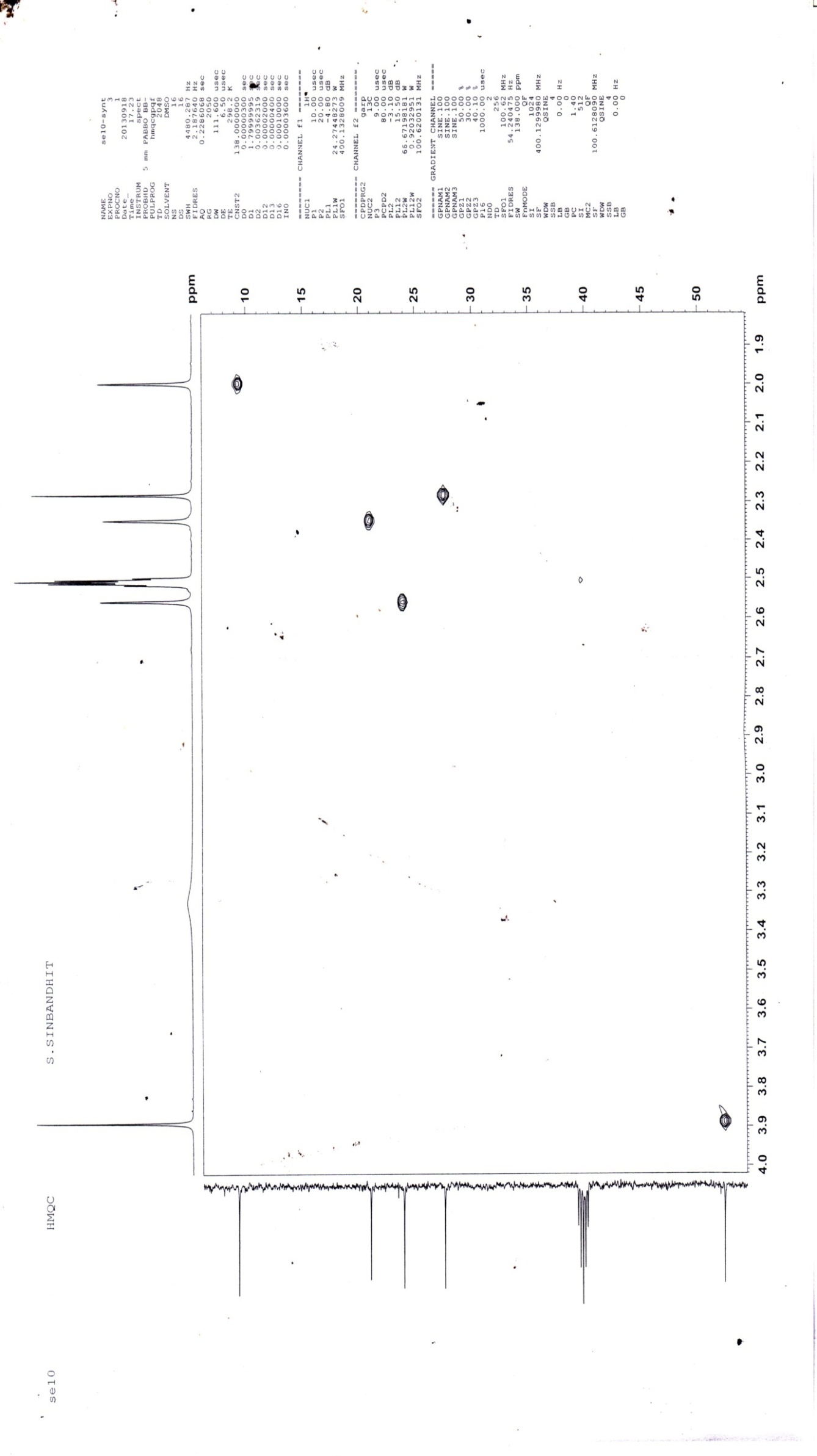


H-8’

C-8’

H-11

C-11

H-9’

C-9’

C-12

H-12

OCH_3_-7’

OCH_3_-7’

**Figure S1D** HMQC spectrum of compound **2**


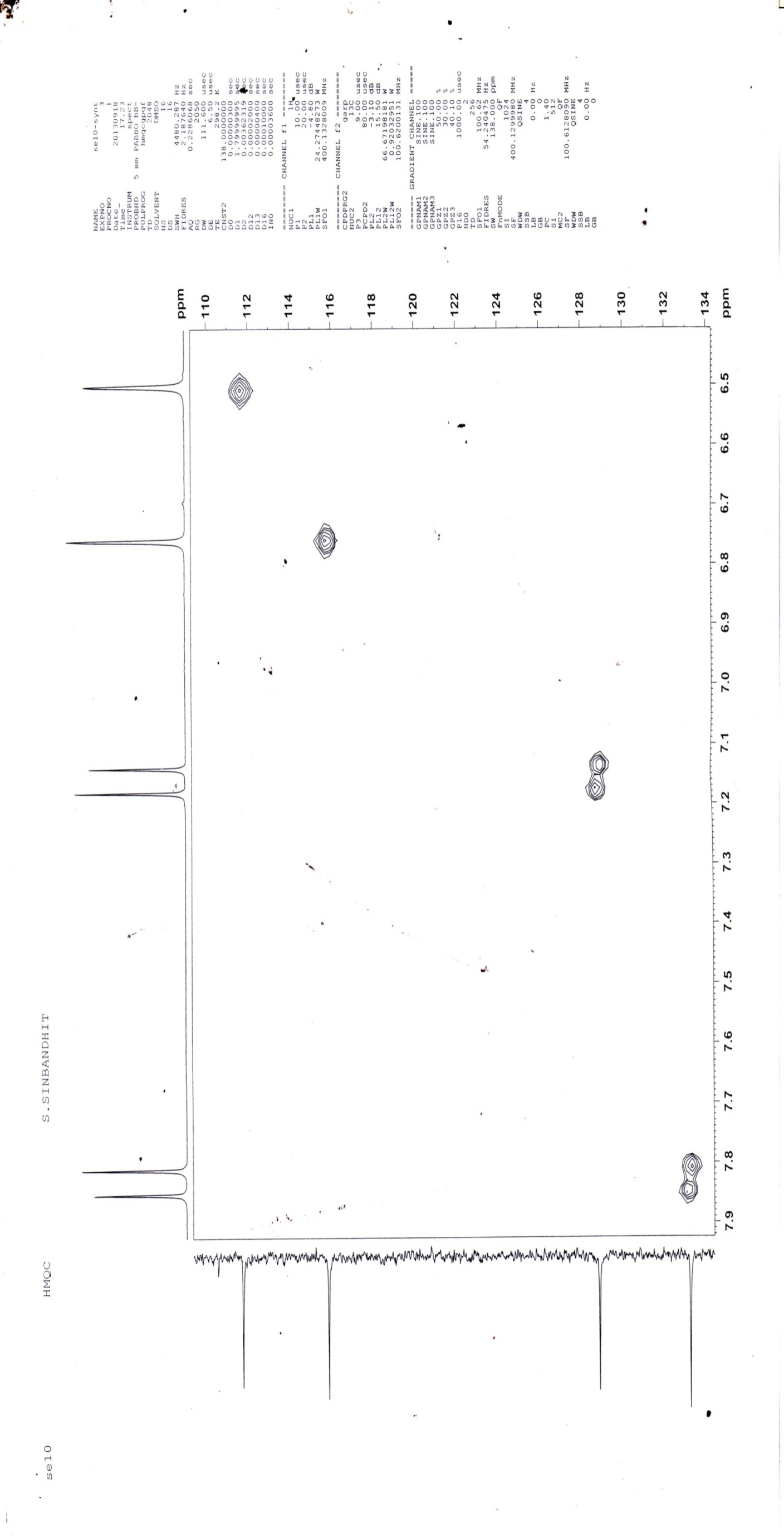


H-5

C-5

H-5’

C-5’

H-9

H-8

C-9

C-8

**Figure S1E** HMQC spectrum of compound **2**


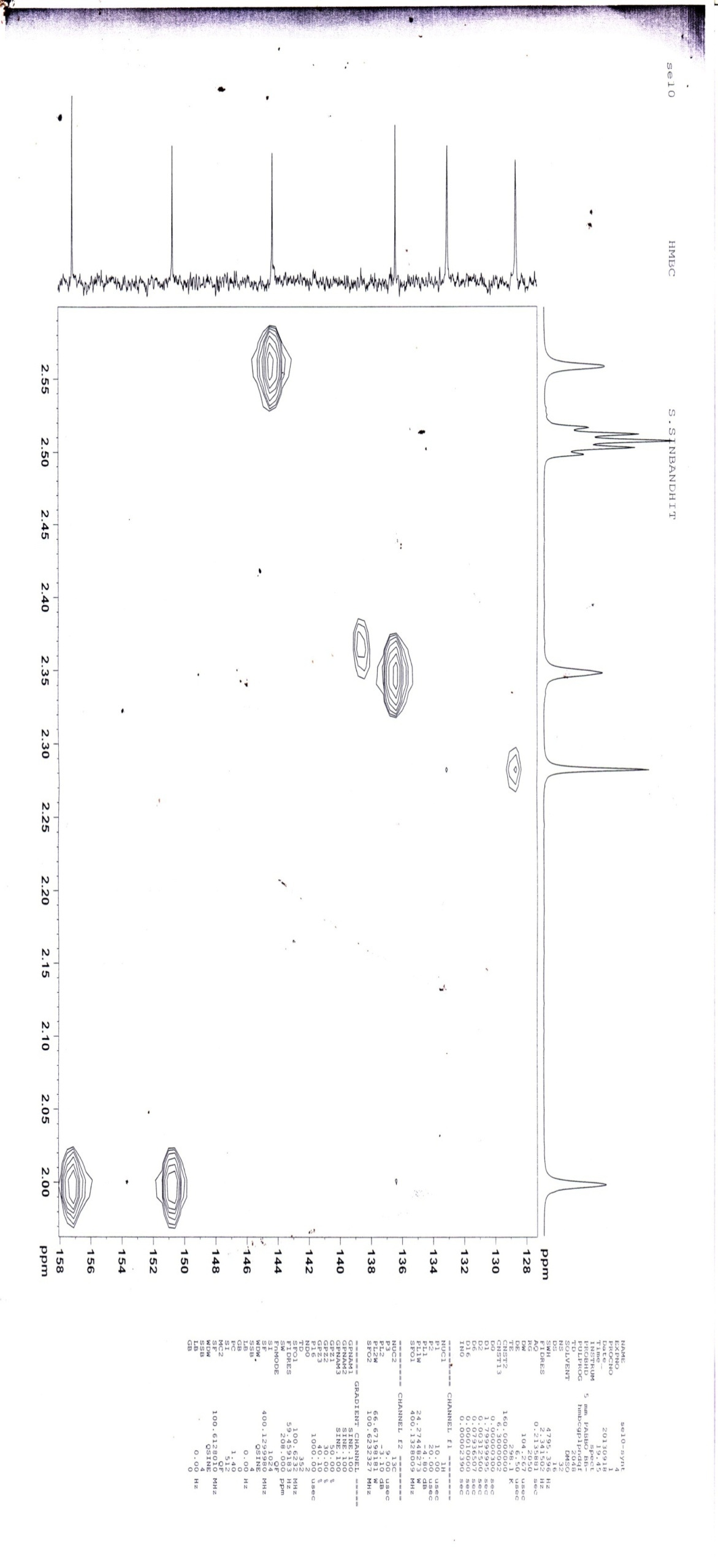


H-8’

C-9

H-9’

C-8

H-11

H-12

C-2’

C-6’

C-6

C-4’

**Figure S1F** HMBC spectrum of compound **2**


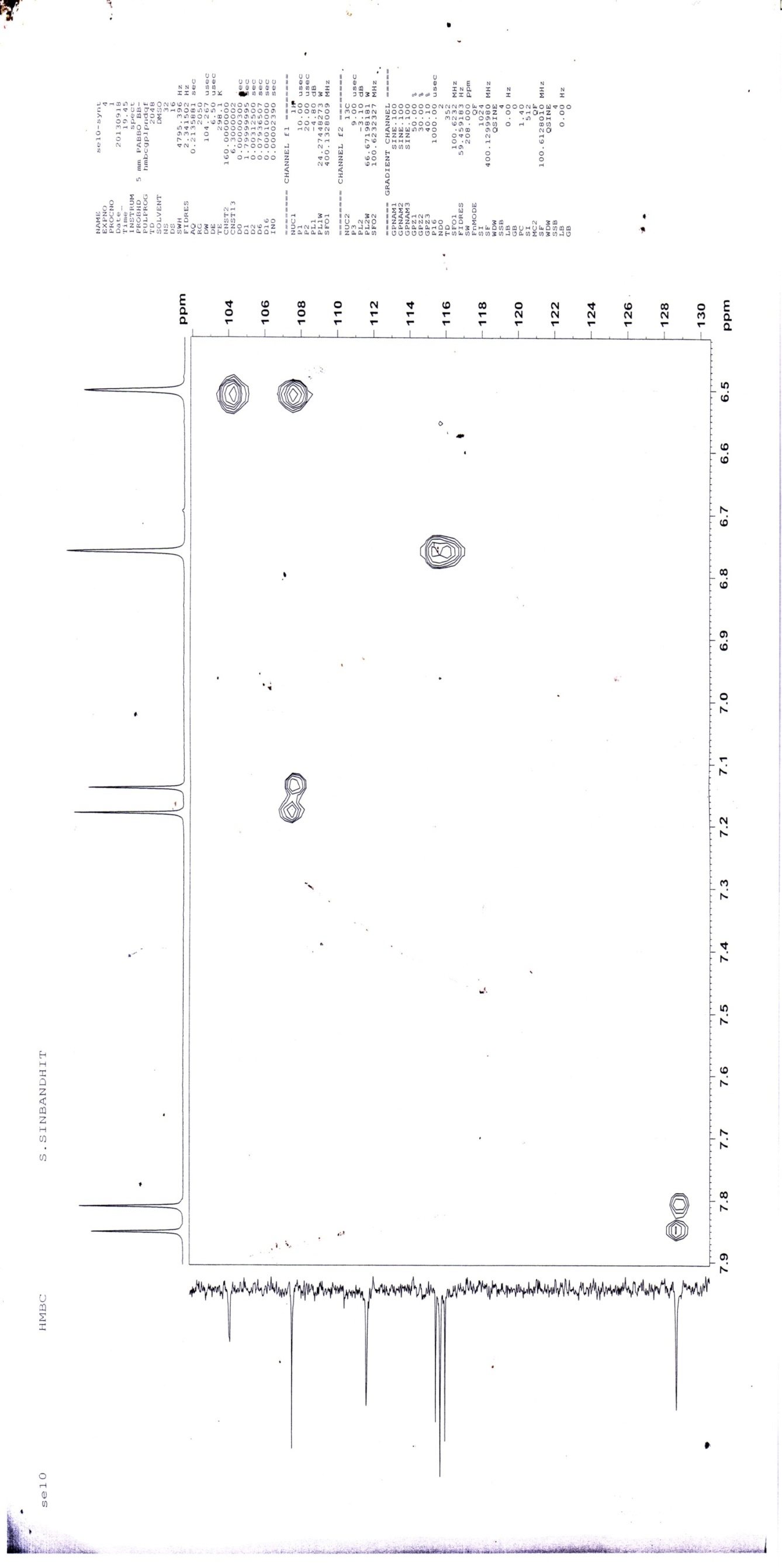


H-5

C-1

H-5’

C-3

H-9

H-8

C-9

C-5

C-1’

C-5’

C-3’

**Figure S1G** HMBC of compound **2**


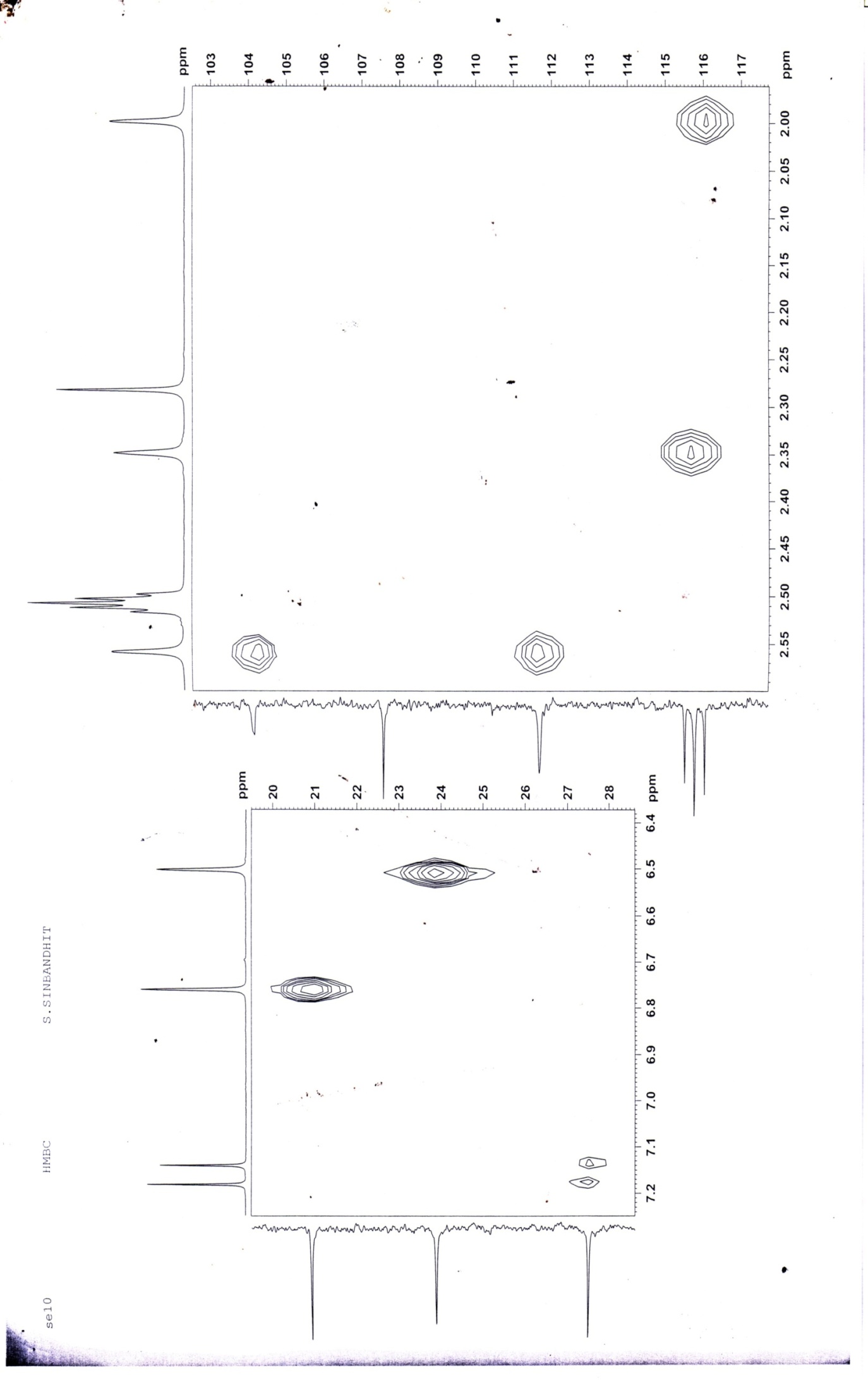


H-8’

C-9’

H-11

C-12

H-9’

H-5’

C-1’

C-1

C-3

C-11

C-5

H-12

H-5

H-9

C-5’

C-3’

**Figure S1H** HMBC of compound **2**
