# Supplementary figures and images for: Depsides: Lichen Metabolites Active against Hepatitis C Virus
Source: PLoS One. 2015 Mar 20;10(3):e0120405. doi: 10.1371/journal.pone.0120405 (PMC4368788; doi:10.1371/journal.pone.0120405)

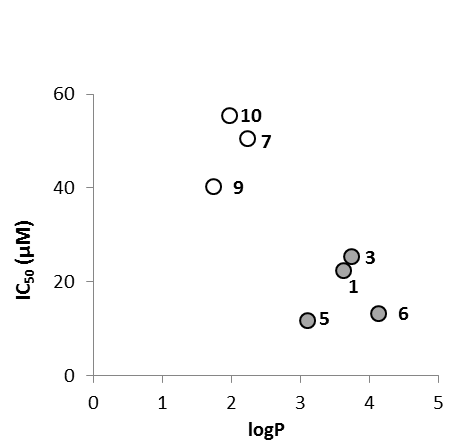

Supplement: S3 Fig — One day after seeding at 50,000 cells/cm2, Huh-7.5.1 cells were incubated 48 h with HCVcc at a MOI ~ 0.03 in the presence of various concentrations of the tested compounds which were already added to cell culture 1 h before. Viral replication was assessed at the end of the incubation periods to determine the respective IC50 (mean ± SEM, n = 3, see Table 2 in the main manuscript) of each compounds. The antiviral activities (IC50) of lichen metabolites (depsides in gray circles and monoaromatic phenols in open circles) were represented according to their theoretical partition-coefficients (logP) predicted with the free software ALOGPS 2.1. For clarity, three lichen metabolites were excluded from the analysis: compound 2 for its inaccurate IC50 value due to its instability, and the inactive compounds 4 and 8. (DOC) [file pone.0120405.s003.doc]
