# Supplementary material for: Depsides: Lichen Metabolites Active against Hepatitis C Virus
Source: PLoS One. 2015 Mar 20;10(3):e0120405. doi: 10.1371/journal.pone.0120405 (PMC4368788; doi:10.1371/journal.pone.0120405)
Supplement: S1 Protocol — (DOC) [file pone.0120405.s004.doc]

**File S1. Detailed protocol for extraction and isolation of lichen metabolites**

Air-dried thalli of the lichen *S. evolutum* Graewe (300 g) were successively extracted with *n*-hexane, acetone and tetrahydrofuran by maceration for one day (3 times x 2 L) at room temperature. A pure white compound **1** (6.20 g) precipitated from the *n*-hexane and the acetone extracts at room temperature. Concentration of the acetone filtrate under vacuum until 50 ml, followed by filtration at room temperature yielded a white precipitate P1 (1.55 g) and a soluble portion S1 (4.78 g). Fraction P1 was purified by using flash chromatography on C_18_ (RS 40 C_18_ ec) eluted with a water-acetonitrile gradient system (9:1, 8:2, 7:3, 6:4, 5:5, 4:6, 0:1, each 200 mL), to give fractions Fa1 - Fa7. Compound **11** (stictic acid) precipitated as an amorphous white solid (25.5 mg) in fraction Fa3 while compound **13** (lobaric acid) was obtained from the supernatant of the fraction Fa6 as a white crystalline solid after recristallization in acetone (1.2 g).

The *n*-hexane filtrate (1.55 g) was chromatographed on a silica gel column (3,5 x 40 cm) by gradient elution with *n*-hexane in methanol (MeOH) system (1:0 to 0:1, each 100 mL) through ethyl acetate (EtOAc) to obtain ten subfractions (Fb1-Fb10). Fraction Fb4 (77 mg, *n*-hexane-EtOAc 9:1), was subjected to column (2.0 x 25 cm) chromatographic separation over silica gel (15 g) and eluted with toluene-EtOAc (75:25, 400 mL) as the mobile phase to give compound **15** (7.0 mg) (brassicasterol). Fraction Fb6 (97 mg, *n*-hexane-EtOAc (8:2)), was further purified by circular chromatography (*n*-hexane-dichloromethane (3:7), 120 mL), then on a silica gel column chromatography (2 x 30 cm) using cyclohexane-EtOAc (75:25, 200 mL) to afford compound **16** (8.5 mg) (ursolic acid). Fraction Fb7 (60 mg, *n*-hexane-EtOAc (7:3)) was fractionated by a silica gel column chromatography (2 x 25 cm) using *n*-hexane-dichloromethane (CH_2_Cl_2_) (8:2, 7:3, 6:4, 5:5, each 50 mL) as eluent and a white precipitate was formed in fraction eluted with *n*-hexane-CH_2_Cl_2_ (6:4), then separated from the supernatant by centrifugation to yield compound **17 (**23.3 mg,). The acetone filtrate S1 (4.8 g) was fractionated on a C18 flash column (Chromabond® Flash, RS 40 C_18_ ec; Sorbent 43g C_18_ ec, 71 mL, conditioning volume 110 mL) using a step gradient of H_2_O-acetonitrile (CAN) 9:1, 8:2, 7:3, 6:4, 5:5, 0:1 (10 mL/min, each 375 mL), then acetone (100%, 200 mL) to give 11 subfractions (Fc1-Fc11). Compound **4** (5.2 mg) (Cladonioidesin) was obtained from fraction Fc3 (240 mg, H_2_O-ACN (7:3)) by silica gel column chromatography (2 x 30 cm) using diethylether-MeOH (85:15, each 15 mL). Fraction Fc4 (398 mg, H_2_O:ACN (6:4)) was chromatographed on a silica gel column (2 x 30 cm) using a solvent gradient *n*-hexane-CH_2_Cl_2_ (100:0 → 0:100) as eluent, to yield 6 fractions (Fc4a-Fc4f). Compound **7** (159 mg) (MOC) precipitated as a white crystalline in fraction Fc4a (*n*-hexane:CH_2_Cl_2_ (2:8)). Fraction Fc4b (44.1 mg, *n*-hexane:CH_2_Cl_2_ (1:9)) was further subjected to column chromatography (1.0 x 20 cm) with toluene-EtOAc (7:3, 300 mL, 5 mL fractions) to give compound **8** (6.5 mg) and compound **9** (9.9 mg). Fraction Fc5 (90 mg, H_2_O:ACN (6:4)) was subjected to column (2 x 25 cm) chromatographic separation over silica gel eluting with a *n*-hexane-chloroform (CHCl_3_) gradient system (8:2, 7:3, 6:4, 5:5, 2:8, 0:1, 100 mL) to give compound **14** with *n*-hexane:CHCl_3_ (2:8) (5 mg). Fraction Fc6 (395 mg, H_2_O:ACN (5:5)) was subjected to silica gel column chromatography (2 x 30 cm), eluting with *n*-hexane-CH_2_Cl_2_ (8:2, 9:1, 0:1), then CH_2_Cl_2_:acetone (0:1 → 1:0) to give nine subfractions Fc6a – Fc6i. Compound **12** (7.2 mg) (isidiophorin) was obtained from fraction Fc6e (10.4 mg, CH_2_Cl_2_:Me_2_CO (6:4)) by prepacked C_18_ column chromatography (Chromabond®, ACN:H_2_O, 6:4, 300 mL). Fraction Fc8 (493 mg, H_2_O:ACN (0:1)) was chromatographed on a silica gel column (2.5 x 40 cm) using *n*-hexane-CH_2_Cl_2_ (2:8, 1:9, 0:10, each 100 mL), then an increasing gradient solvent system of EtOAc in CH_2_Cl_2_ (0-100% EtOAc with 10% increment for each step; 100 mL each) to afford nine subfractions Fc8a-Fc8i. Compound **2** (8.4 mg) was obtained from supernatant of fraction Fc8d (CH_2_Cl_2_:EtOAc (8:2)). Compound **10** (14.5 mg) (Methyl haematommate) was obtained from fraction Fc9 (63 mg, H_2_O:ACN (0:1)) chromatographed on a silica gel column (2 x 25 cm) using *n*-hexane-EtOAc (95:5, 500 mL). Fraction Fc11 (1.22 mg, acetone 100%) was fractioned on a silica gel column (2.5 x 40 cm) with a gradient solvent system of a mixture of *n*-hexane-EtOAc, through CHCl_3_, beginning with *n*-hexane-CHCl_3_ 7:3 to CHCl_3_:EtOAc (0:1) and produced eight fractions (Fc11a-Fc11h). Fraction Fc11b (173 mg, *n*-hexane-CHCl_3_ (1:9)) was further subjected to silica gel flash chromatography (Chromabond® flash, RS 15 SiOH) using a gradient solvent system of CH_2_Cl_2_-MeOH (100:0 → 95:5, 10 mL/min, 500 mL, each 10 mL). Fraction 17-22 (40.8 mg, solvent-proportion) was then purified by a silica gel column (1.5 x 25 cm) using *n*-hexane-CH_2_Cl_2_ (8:2, 300 ml,) to yield compound **3** (20,9 mg) (Methyl-3’-methyl lecanorate).

Known compounds were identified by comparison of their physical and spectra data with the published values.

***Compound*** (**2)***:* White powder; m.p. 234-236^0^C (dec); UV (Acetonitrile/tetrahydrofuran) λ_max_ (log ε) = 278 (4.80), 306 (4.64); IR ν_max_ = 3147, 1653, 1610, 1580, 1441, 1396, 1257,1150 cm^-1^; HR-ESI-MS: m/z = 413.1237 [M-H]^-^ (calcd for C_22_H_21_O_8_: 413.1241); ^1^H NMR and ^13^C NMR see Table 1.
