# Supplementary material for: Depsides: Lichen Metabolites Active against Hepatitis C Virus
Source: PLoS One. 2015 Mar 20;10(3):e0120405. doi: 10.1371/journal.pone.0120405 (PMC4368788; doi:10.1371/journal.pone.0120405)
Supplement: S1 Table — (DOCX) [file pone.0120405.s005.docx]

| **Table S1.** **Statistical comparison of anti-HCV activity of lichen metabolites**. | | | | | | | | | | |
| --- | --- | --- | --- | --- | --- | --- | --- | --- | --- | --- |
|  |  | | |  | |  |  | | |  |
|  | **Compound**  **comparison** | | | **Mean**  **difference** ^a^ | **q** | | | **P Value** ^b^ | | |
| **Intra-**  **depsides** | **1** | vs | **3** | -3.1 | - | | | ns | | |
|  | **1** | vs | **5** | 10.5 | - | | | ns | | |
|  | **1** | vs | **6** | 9.0 | - | | | ns | | |
|  | **3** | vs | **5** | 13.6 | 2.874 | | | ns | | |
|  | **3** | vs | **6** | 12.2 | - | | | ns | | |
|  | **5** | vs | **6** | -1.4 | - | | | ns | | |
|  |  |  |  |  |  | | |  |  | |
| **Intra-**  **Monoaromatic phenols** | **7** | vs | **9** | 10.3 | - | | | ns | | |
|  | **7** | vs | **10** | -4.9 | - | | | ns | | |
|  | **9** | vs | **10** | -15.2 | 3.217 | | | ns | | |
|  |  |  |  |  |  | | |  |  | |
| **Inter-**  **depsides-**  **Monoaromatic phenols** | **1** | vs | **7** | -28.3 | 5.975 | | | ** | P<0.01 | |
|  | **1** | vs | **9** | -18.0 | 3.799 | | | * | P<0.05 | |
|  | **1** | vs | **10** | -33.2 | 7.016 | | | ** | P<0.01 | |
|  | **3** | vs | **7** | -25.1 | 5.312 | | | ** | P<0.01 | |
|  | **3** | vs | **9** | -14.8 | 3.136 | | | * | P<0.05 | |
|  | **3** | vs | **10** | -30.1 | 6.353 | | | ** | P<0.01 | |
|  | **5** | vs | **7** | -38.8 | 8.186 | | | *** | P<0.001 | |
|  | **5** | vs | **9** | -28.5 | 6.010 | | | ** | P<0.01 | |
|  | **5** | vs | **10** | -43.7 | 9.227 | | | *** | P<0.001 | |
|  | **6** | vs | **7** | -37.3 | 7.882 | | | *** | P<0.001 | |
|  | **6** | vs | **9** | -27.0 | 5.707 | | | ** | P<0.01 | |
|  | **6** | vs | **10** | -42.2 | 8.924 | | | *** | P<0.001 | |
| ^a^Difference between respective IC_50_. ^b^Statistical significance of differences was assessed using GraphPad InStat 3 software. An ANOVA followed by a Student-Newman-Keuls test was used to compare the IC_50_ of active molecules. Three lichen metabolites were excluded from the analysis: compound **2** for its inaccurate IC_50_ value due to its instability, and the inactive compounds **4** and **8**. | | | | | | | | | | |
